# Cross-talk between down-regulation of steroidogenic genes expression and oxidative and apoptotic biomarkers in testes induced by administration of tramadol and boldenone and their combination in male albino rats

**DOI:** 10.22038/IJBMS.2022.61745.13662

**Published:** 2022-07

**Authors:** Noha A. Mowaad, Gihan F. Asaad, Marwa E.A. El-Shamarka, Sahar Khalil

**Affiliations:** 1 Department of Narcotics, Ergogenics, and Poisons, Medical Research and Clinical Studies Institute, National Research Centre, Cairo, Egypt; 2 Department of Pharmacology, Medical Research and Clinical Studies Institute, National Research Centre, Cairo, Egypt; 3 Department of Histology, Faculty of Medicine, Suez Canal University, Ismailia, Egypt

**Keywords:** Boldenone, HSD17B3, Infertility, ROS, StaR, Steroids, Testes, Tramadol

## Abstract

**Objective(s):**

The testis is the male reproductive gland or gonad having two vital functions: to produce both sperm and androgens, primarily testosterone. The study aimed to investigate the effect of tramadol and boldenone injected alone or in combination for 2 months in rats on testicular function.

**Materials and Methods:**

Group 1, normal control; Group 2, tramadol HCl (TRAM) (20 mg/kg bwt.) (IP); Group 3, boldenone undecylenate (BOLD) (5 mg/kg bwt) (i.m); Group 4, combination of TRAM (20 mg/kg bwt.) and BOLD (5 mg/kg); respectively for 2 months.

**Results:**

TRAM and BOLD alone and in combination showed deteriorated testicular functions, lowered serum steroid levels (FSH, LH, and testosterone), elevation in oxidative biomarkers (MDA & NO) and reduction in GSH and SOD, down-regulation of StaR and HSD17B3 as well as histopathological testicular assessment using H&E staining revealing massive degenerative changes in the seminiferous epithelium and vacuolar changes of most of the spermatogenic stages in both TRAM and BOLD groups. PAS stain showed an intensive reaction in the interstitial tissue between the tubules in the TRAM group. Masson trichrome stain showed abundant collagen fiber deposits in the tunica albuginea with congested BV in the TRAM group.

**Conclusion:**

The study illuminated the hazard of administration of these drugs for a long period as well as the prominent deleterious effects reported on concurrent use of both drugs.

## Introduction

One of the vital functions of the testis is the production of androgens, primarily testosterone. Testosterone enhances the arising of the distinctive male phenotype as well as the production of sperm. This biological function is under the control of luteinizing hormone (LH) and follicle-stimulating hormone (FSH) released from the anterior lobe of the pituitary gland. LH acts on the Leydig cells to enhance testosterone production while FSH targets the Sertoli cells to enhance the production of androgen-binding protein to which testosterone will bind, localized in high concentrations near the sperm helping normal spermatogenesis (1). Leydig cells by LH, cholesterol is mobilized to the inner mitochondrial membrane utilizing steroidogenic acute regulatory protein (StAR) which is encoded by the StAR gene (2). Inside the inner mitochondrial membrane, cholesterol will be metabolized by cytochrome P450 to pregnanolone, then the pregnanolone will diffuse out of the mitochondria to the smooth endoplasmic reticulum where it is converted to progesterone by 3β-hydroxysteroid dehydrogenase 1 (HSD3B1, encoded by Hsd3b1 gene). Finally, progesterone will be converted into testosterone through cytochrome P450 17α-hydroxylase/C17–20 lyase (CYP17A1, encoded by Cyp17a1) and 17β-hydroxysteroid dehydrogenase isoform 3 (HSD17B3, encoded by Hsd17b3 gene) (3).

Oxidative stress occurred due to imbalance between the antioxidant defense mechanisms and reactive oxygen species (ROS) production (4). ROS are unstable and very reactive by-products released during normal metabolism, leading to damage to the main biomolecules (4). To the best of our knowledge, testosterone enhances the metabolic rate (5), leading to the production of sexual ornaments, and so alters the balance between ROS production and antioxidant defenses, resulting in enhanced risk of oxidative stress. On the other hand, there is a direct relation between the increase in ROS production and reduction in testosterone levels (6). According to von Schantz *et al*. (7), many studies have assumed that testosterone possesses pro-oxidant properties (8), even though a closer examination of available data reveals a more multifaceted pattern. High testosterone levels produce oxidation in rat testicular tissues (9), but testosterone also possesses antioxidant properties in the human prostate (10) and rat nervous system (11). These findings, therefore, revealed that the pro-oxidant effect of testosterone depends on gender and the type of tissues affected.

Tramadol is a centrally acting analgesic used in the treatment of moderate acute or chronic pain but it is believed to be only a weak μ- receptor agonist and later on, its predominant mechanism of action showed to be based on blockade of serotonin reuptake as well as stimulation of D2 receptor which results in increased activity of dopamine (12). This effect endorsed tramadol to be abused in the Egyptian community which may, unfortunately, develop drug tolerance after prolonged administration of tramadol (13). Besides, tramadol is used as an effective form of treatment for mild to severe premature ejaculation at therapeutic doses (14). Generally, endogenous and exogenous opioids, modulate the gonadotropin-releasing hormone by targeting the opioid receptors in the hypothalamus and so reducing the LH and FSH release that have a direct effect on testosterone production in testes (15). 

Boldenone is a well-known anabolic androgenic steroid (AAS) that is administered by bodybuilders and athletes to develop their physical and muscular performance. This effect takes place by enhancing protein anabolism and reducing protein catabolism in addition to water, nitrogen, and electrolyte retention (16). Generally, anabolic androgenic steroids develop cardiovascular system dysfunction after prolonged use (17). Endocrine impairment has also been reported via triggering negative feedback on the hypothalamic-pituitary axis leading to inhibition of LH and FSH (18). Testicular atrophy and gynecomastia are commonly reported after administration of AAS (19). 

Nowadays the use of opioids and AAS are noticeably increasing which may lead to severe damage to the testes as well as infertility in males if they are administered in combination. In the current study, the effect of chronic administration of tramadol and boldenone alone or in combination for 2 consecutive months on the testicular tissue has been assessed by calorimetric measuring of oxidative biomarkers such as malondialdehyde (MDA), reduced glutathione (GSH), superoxide dismutase (SOD) and nitric oxide (NO). The apoptotic effect was also determined by ELISA detection of B-cell lymphoma 2 (Bcl2), Bcl2 Associated X, Apoptosis Regulator (Bax), Bax/Bcl2 ratio, and caspase3 concentrations. The hormonal changes of LH, FSH, and testosterone have been reported in serum as well as PCR quantification for the first time of both StAR and HSD17B3 gene expression that regulates the production of testosterone in rat testicular tissue. 

## Materials and Methods

***Animals***

Thirty-two male Wistar albino rats (250–350 g) were purchased from the animal breeding unit at the National Research Centre (Dokki, Giza, Egypt). Animals were housed in cages with water and food *ad libitum*, and the animal room temperature was kept at a constant temperature of 20 ± 1 °C on a 12-hr light/12-hr dark cycle. Adequate measures were taken to minimize the pain and discomfort of the animals. Experimental protocol followed the Ethics and Animal Care Committee of the National Research Centre and the recommendations of the National Institutes of Health Guide for Care and Use of Laboratory Animals (NIH Publications No. 8023, revised 1978). 

***Drugs and reagents***

Tramadol tablets were officially provided by the Ministry of Justice ( Egypt ) and dissolved in saline. Boldenone undecylenate (50 mg/ml in sesame oil) was purchased from Tornel S.A. Laboratories, Mexico. Other chemicals were of the highest commercial grade available.

***ELISA kits***

Rat B-cell CLL/lymphoma 2 (BCL2) ELISA kit (China-CUSABIO, Catalog number CSB-E08854r). Rat Apoptosis regulator BAX(BAX) ELISA kit (China- CUSABIO, Catalog number CSB-EL002573RA). Rat Caspase 3, Casp-3 ELISA Kit (China- CUSABIO, Catalog number CSB-E08857r). Rat luteinizing hormone (LH) Elisa kit (China-CUSABIO, Catalog number CSB-E12654r). Rat follicle-stimulating hormone, FSH ELISA Kit (China-CUSABIO, Catalog number CSB-E06869r). Rat Testosterone ELISA Kit (China-CUSABIO, Catalog number CSB-E05100r).

***Experimental protocol***

Thirty-two rats were randomly divided into four groups (n=8 per group); Group 1: kept as the control group, received saline (IP) daily and sesame oil (IM) once weekly for 60 days, Group 2: was injected tramadol (20 mg/kg) daily intraperitoneally (IP) (20) for 60 days, Group 3: was injected boldenone undecylenate (5 mg/kg) (IM) once weekly for 60 days week (21), and Group 4 was injected a combination of tramadol intraperitoneally (20 mg/kg.) and boldenone undecylenate (5 mg/kg) (IM) once weekly for 60 days week. All groups were treated for 2 months. On the 60^th^ day, blood samples were taken from the retroorbital plexus under light anesthesia according to Van Rijn *et al*. (22).

For estimation, hormonal levels in serum (LH, FSH, and Testosterone) were assessed using ELISA kits and according to the manufacturer’s instructions. Animals were then sacrificed by cervical dislocation under anesthesia and both testes from all rats were extracted. One testis was kept at -80 °C for colorimetric determination of malondialdehyde (MDA), reduced glutathione (GSH), superoxide dismutase (SOD), and nitric oxide (NO). Bcl2, Bax, and caspase-3 (Casp-3) were determined using the ELISA technique. StAR and HSD17B3 genes were quantified in tissue using real-time polymerase chain reaction (PCR). The other testis was kept in formalin 10% for histopathological and histochemical studies.

***Preparation of tissue samples***

The frozen testis from each rat from all the groups were homogenized (MPW-120 homogenizer, Med instruments, Poland) in PBS to obtain 20% homogenate and kept overnight at –80 °C. The homogenates were centrifuged for 5 min at 5000 x g using a cooling centrifuge (Sigma and laborzentrifugen, 2k15, Germany). The supernatant was taken immediately and used for oxidative stress biomarkers (MDA, GSH, SOD, and NO) using colorimetric kits according to the following methods (23–26). Apoptotic markers Bcl2, Bax, and Caspase-3 (Casp-3) using specific ELISA kits according to the manufacturer’s instructions. All results are calculated as 1mg total protein.

***Detection of gene expression of StAR and HSD17B3 in testicular tissue by Quantitative real-time polymerase chain reaction (qRT-PCR)***

Total RNA was isolated using a Qiagen tissue extraction kit (Qiagen, USA) according to the instructions of the manufacturer. The total RNA was used for complementary DNA (cDNA) conversion using high-capacity cDNA reverse transcription kit (Fermentas, USA). Moloney murine leukemia virus (MMLV) reverse transcriptase was used for synthesis of cDNA from RNA. Human Placental Ribonuclease Inhibitor (HPRI) was used for inhibition of RNase activity. Real-time qPCR amplification and analysis were performed using an Applied Biosystem with software version 3.1 (StepOne™, USA). The reaction contained SYBR Green Master Mix (Applied Biosystems), gene-specific primer pairs which were shown in Table 1 and were designed with Gene Runner Software (Hasting Software, Inc., Hasting, NY, USA) from RNA sequences from the gene bank. All the primer sets had a calculated annealing temperature of 60 °C. Amplification conditions were: 2 min at 50 °C, 10 min at 95 °C, and 40 cycles of denaturation for 15 sec and annealing/extension at 60 °C for 10 min. The relative expression of the studied genes was calculated according to Applied Biosystem software using the comparative threshold cycle method. All values were normalized to the (beta-actin) genes as an endogenous control (reference gene).

***Histological and histochemical samples***

The other testis was removed and fixed in 10% neutral buffered formalin for 24 hr, then dehydrated using a series of ascending concentrations of alcohol up to 100% alcohol, cleared in two changes of xylol, and embedded in melted paraffin wax. Sections of 5 μm thickness were cut using a rotatory microtome. For histological examination, sections were stained by:

*Hematoxylin and Eosin (H&E) stain to assess the changes in the histological structure of the seminiferous tubules, degenerative and necrotic changes in the spermatogenic cells, and interstitial cells of Leydig.

*PAS stain for histochemical assessment of polysaccharides and glycoproteins in the testes.

*Masson’s trichrome stain to assess the changes in the collagen fibers. Once stained, the sections were dehydrated with ethanol, cleared in xylene, and mounted with “DPX” (27). 

All parameters were assessed in 6 randomly selected non-overlapping fields per tissue section of each sample at a magnification of X400 using the image analyzer computer system (Leica Microsystems GmbH, Germany) operated by Leica Application software for tissue section analysis. The optical density of both the positive PAS-stained material and collagen fibers was calculated.

***Statistical analysis***

Data were represented as mean±SE. Statistical analysis was carried out by one-way analysis of variance (ANOVA) followed by the Tukey-Kramer test for multiple comparisons. GraphPad Prism software version 8.4.3 (GraphPad Software, Inc.) was used for data analysis. Values of *P*<0.05 were considered significant.

## Results

***Assessment of oxidative stress biomarkers***

Animal groups were injected intraperitoneally with tramadol 20 mg/kg and intramuscular with boldenone 5 mg/kg, and their combination for 2 months showed a significant elevation in MDA and NO concentrations (*P*<0.05), when compared with the control group no significant difference occurred between groups injected with tramadol and boldenone. A noticeable significant increase (*P*<0.05) was determined in group 4 injected with a combination of tramadol 20 mg/kg + boldenone 5 mg/kg when compared with groups administered tramadol or boldenone alone. 

Animal groups injected intraperitoneally with tramadol 20 mg/kg and intramuscular boldenone 5 mg/kg and their combination for 2 months showed a significant (*P*<0.05) decrease in GSH and SOD concentrations; when compared with the control group no significant difference occurred between groups injected with tramadol and boldenone. A noticeable significant decrease (*P*<0.05) was determined in group 4 injected with a combination of tramadol 20 mg/kg + boldenone 5 mg/kg when compared with groups administered tramadol or boldenone alone. Results are represented in Table 2.

***Assessment of apoptotic biomarkers***

Bax is an apoptotic protein while Bcl-2 is an anti-apoptotic protein, and the ratio of those proteins determines the occurrence of cell apoptosis. Caspase-3 is also one of the major effectors of apoptosis, and its activation of caspase-3 indicates irreversible cell apoptosis. Animal groups injected intraperitoneally with tramadol 20 mg/kg and intramuscular boldenone 5 mg/kg and their combination for 2 months showed a significant (*P*<0.05) increase in Bax concentrations when compared with the control group. No significant difference occurred between groups injected with tramadol and boldenone. A noticeable significant increase (*P*<0.05) was determined in the group 4 injected with a combination of tramadol 20 mg/kg + boldenone 5 mg/kg when compared with groups administered tramadol or boldenone alone. On the other hand, animal groups injected intraperitoneally with tramadol 20 mg/kg and boldenone 5 mg/kg and their combination for 2 months showed a significant (*P*<0.05) decrease in Bcl2 concentrations when compared with the control group. No significant difference occurred between groups injected with tramadol and boldenone. A noticeable significant decrease of *P*<0.05 was determined in the group 4 injected with a combination of tramadol 20 mg/kg + boldenone 5 mg/kg when compared with groups administered tramadol or boldenone alone. Bax/Bcl2 ratio acts as a marker for the determination of cell susceptibility to apoptosis. Groups injected with tramadol, boldenone, and their combination showed a remarkable increase in Bax/Bcl2 ratio; we can notice that Bax/Bcl2 ratio for groups given tramadol and boldenone showed nearly the same ratio while group 4 injected with the combination showed a significantly higher ratio than both group 2 and 3. Caspase-3 is another apoptotic biomarker that showed the same results as those of Bax where we found that the groups injected intraperitoneally with tramadol 20 mg/kg and boldenone 5 mg/kg and their combination for 2 months showed a significant (*P*<0.05) increase in Casp -3 concentrations when compared with the control group. No significant difference occurred between groups injected with tramadol and boldenone. A noticeable significant increase (*P*<0.05) was determined in group 4 injected with a combination of tramadol 20 mg/kg + boldenone 5 mg/kg when compared with groups administered tramadol or boldenone alone. Results are represented in Table 3.

***Determination of hormonal changes in serum***

Animal groups injected intraperitoneally with tramadol 20 mg/kg for 2 months showed a significant (*P*<0.05) decrease in LH, FSH, and testosterone, as compared with the control group, intramuscular injection of boldenone 5 mg/kg for 2 months showed a significant (*P*<0.05) decrease in concentrations of LH, FSH, and testosterone in serum respectively, as compared with control group and with the group injected with tramadol alone. The group injected with a combination of tramadol 20 mg/kg + boldenone 5 mg/kg showed a significant (*P*<0.05) decrease in LH and FSH in serum respectively, as compared with the control group and the group injected with tramadol alone. It was also shown that injection with combination does not show a significant (*P*<0.05) difference in testosterone concentration in serum when compared with the group injected with boldenone only. Results are presented in Table 4.

***Assessment of gene expression of StAR and HSD17B3 in testicular tissue by Quantitative real-time polymerase chain reaction (qRT-PCR)***

**Figure 1:**
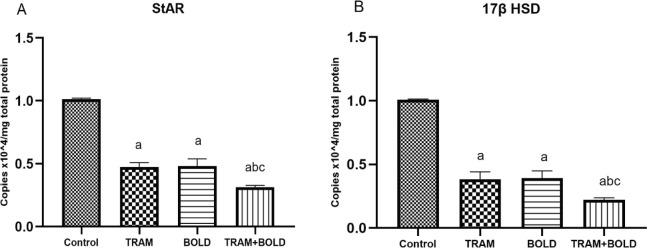
Effect of TRAM, BOLD, and their combination on (A)StAR and (B)17β HSD expression in rat testis. All data are presented as mean ± SE, a *P*-value of < 0.05 was assumed to denote statistical significance. a*P*<0.05 compared with the control group, b*P*<0.05 vs Tram group and c*P*<0.05 vs Bol group

Animal groups injected intraperitoneally with tramadol 20 mg/kg and intramuscular boldenone 5 mg/kg and their combination for 2 months showed a significant (*P*<0.05) decrease in StAR and 17β HSD gene expression (StAR; 0.474±0.04, 0.482±0.07 and 0.314±0.02 Copiesx10^4 /mg protein) and (17β HSD; 0.382±0.08, 0.392±0.07 and 0.22±0.02 Copiesx10^4 /mg protein), respectively, when compared with control group (StAR; 1.014±0.009 Copiesx10^4 /mg protein and 17β HSD; 1.01±0.006 Copiesx10^4 /mg protein). No significant difference occurred between groups injected with tramadol and boldenone. A noticeable significant increase (*P*<0.05) was detected in group 4 injected with a combination of tramadol 20 mg/kg + boldenone 5 mg/kg when compared with groups administered tramadol or boldenone alone. Results are represented in Figures 1a and 1b.

***Histological and histochemical results***

**Figure 2:**
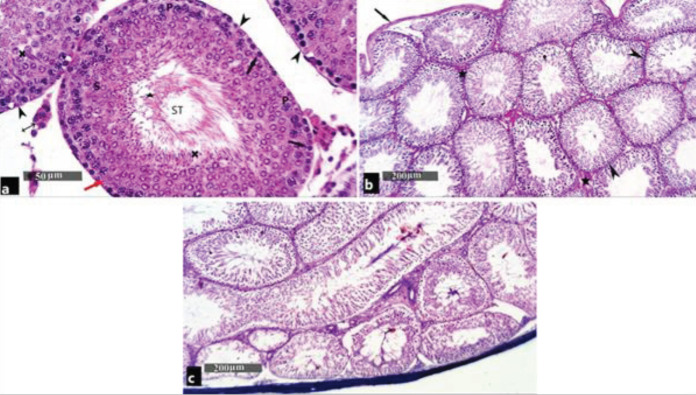
Photomicrographs of testes of control group showing: (a) seminiferous tubules (ST) lined with stratified seminiferous epithelium (S) resting, surrounded by a basal lamina (arrowhead) and lined with spermatogonia (black arrow), primary spermatocytes (P), spermatids (cross), and mature sperms. Mature sperms (star) are seen in the lumen of the Sertoli cells with their triangular nuclei (red arrow) are seen between the spermatogenic cells. The interstitial cells of Leydig (crossed arrow) are seen in between the ST (H&E ×400). (b) Intense positive PAS reaction is seen in the interstitial tissue between the tubules (star) in the capsule (arrow) and the basement membrane (PAS x100). (c) Collagen fibers are seen in tunica albuginea surrounding the testis (Masson's trichrome ×100)

H&E-stained sections of the testes in the control group showed numerous seminiferous tubules that were surrounded by a basement membrane. Each tubule was lined by spermatogenic cells and Sertoli cells. Spermatogenic cells were arranged in many layers starting with spermatogonia next to the basement membrane, followed by primary spermatocytes with their large, rounded nuclei, and condensed chromosomes. Spermatids were noticed as round cells having deeply stained nuclei near the lumen. Meanwhile, the mature sperms were seen in the center of the lumen of the seminiferous tubules. Sertoli cells were seen resting on the basement membrane between the spermatogenic cells with their pyramidal shape and large oval basal nuclei. The tubules are separated from each other by loose interstitial connective tissue containing the Leydig cells with vesicular nuclei (Figure 2a).

**Figure 3:**
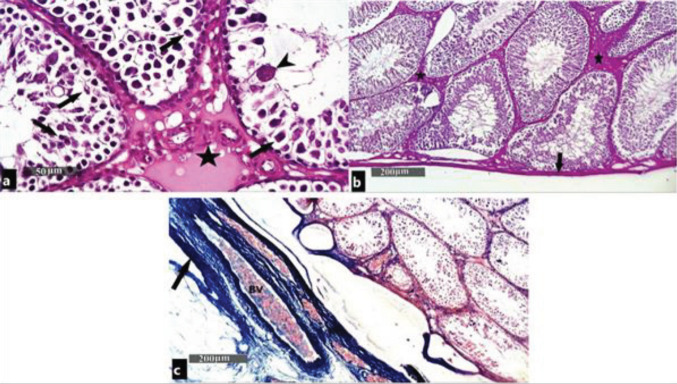
Photomicrographs of testes of the tramadol-treated group showing: (a) Massive degenerative changes in the seminiferous epithelium in the form of nuclear pyknosis and vacuolar changes of most of the spermatogenic stages (arrow). Multinucleated giant cells are seen (arrowhead). The interstitial homogenous material (star) is seen in between seminiferous tubules with congested and dilated blood vessels (H&E ×400). (b) Mild positive PAS reaction is seen in the interstitial tissue between the tubules (star) and in the capsule (PAS x100). (c) Abundant collagen fiber deposits in The tunica albuginea with congested BV and a minimal amount of collagen fibers in the interstitial tissue are seen (Masson's trichrome ×100)

**Figure 4:**
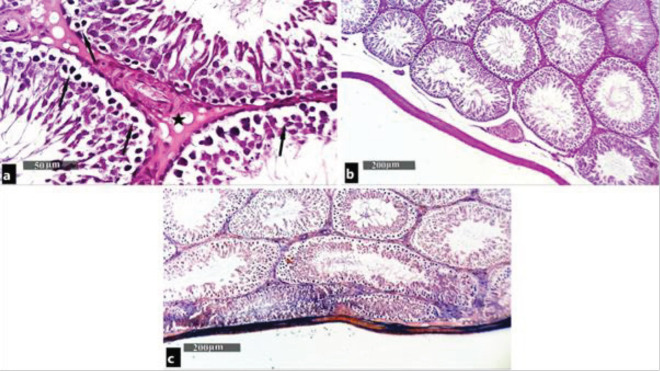
Photomicrographs of testes of the boldenone-treated group showing pictures more or less similar to tramadol treated group. (a) (H&E ×400), (b) (PAS x100), (c) (Masson's trichrome ×100)

**Figure 5:**
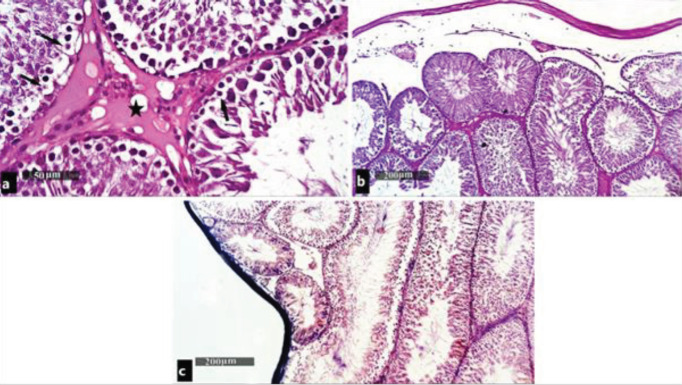
Photomicrographs of testes of tramadol/boldenone-treated group showing no changes in the structure and reactions that are more or less similar to the tramadol group. (a) (H&E ×400), (b) (PAS x100), (c) (Masson's trichrome ×100)

Examination of H&E-stained sections of the testes in the tramadol-treated group showed deformed seminiferous tubules with missing normal histological architecture. Many tubules showed degenerative changes in the form of nuclear pyknosis, cytoplasmic vacuoles, and detached spermatogenic cells. Multinucleated giant cells were also seen in the lumen of the seminiferous tubules with the absence of sperm. Hyalinosed homogenous material and congested blood vessels were also seen in the interstitium (Figure 3a). Examination of H&E-stained sections of the testes in the boldenone-treated group showed nearly the same histological picture with no detectable variation in comparison with the tramadol-treated group (Figure 4a). Examination of H&E-stained sections of the testes in the tramadol/boldenone-treated group showed no detectable changes in the histological picture in comparison with the tramadol-treated group (Figure 5a).

PAS-stained sections of testes in the control group showed PAS-positive reaction in the testicular capsule, basement membrane of the seminiferous tubules, and in the interstitial tissue in between the tubules (Figure 2b). In the tramadol-treated group, boldenone-treated group, and tramadol/boldenone-treated group, mild positive PAS reaction was shown in the interstitial tissue between the tubules and in the testicular capsule. Statistically, the tramadol-treated group, boldenone-treated group, and tramadol/boldenone-treated group showed a significant (*P*<0.05) decrease in PAS-positive material mean optical density (0.1316±0.201, 0.1233±0.193, and 0.1233± 0.193) as compared with the control group (0.2405±0.403). In the meantime, there was no statistically significant difference between the last three groups (Figures 3b, 4b, and 5b).

Masson’s trichrome-stained sections of testes in the control group showed collagen fiber deposition in tunica albuginea (Figure 2c). In the tramadol-treated group, boldenone-treated group, and tramadol/boldenone-treated group, abundant collagen fiber deposits in tunica albuginea and minimal amount of collagen fibers in the interstitial tissue were found.

Statistically, tramadol-treated group, boldenone-treated group, and tramadol/boldenone-treated group showed a significant (*P*<0.05) increase in collagen fiber optical density (0.5123± 0.184, 0.4963± 0.172, and 0.4882± 0.187) as compared with the control group (0.3071± 0.260). In the meantime, there was no statistically significant difference between the last three groups (Figures 3c, 4c, and 5c).

## Discussion

About 50% of the infertility conditions are associated with male factors (28). The main cause of male infertility is still unknown but about 30–80% of the cases were attributed to the release of a high amount of reactive oxygen species (ROS) (29). Many drugs are implicated in infertility disorders due to induction of oxidative stress (30). The testis is considered a susceptible organ to lipid peroxidation due to its high lipid content and high O_2_ consumption (31, 32). This oxidative stress occurs due to an imbalance between ROS and the endogenous antioxidant defense system. Excessive ROS levels lead to detrimental deviations, that can abolish lipids, proteins, nucleic acids, and other cellular compounds, followed by cellular death (33). Consequently, elevated ROS levels in testicular tissue might cause infertility (34).

In the current study, our findings revealed that intraperitoneal injection of tramadol (20 mg/kg) to male rats for 2 months increased the level of oxidative stress evidenced by elevated MDA and NO and on the other hand a significant decrease in GSH and SOD levels in testicular tissue. Accordingly, we concluded that oxidative stress is well-thought to be a principal factor in the induction and progression of tramadol-induced reproductive toxicity (35). Our findings were in agreement with the prior studies reported by Abdel-Latif Ibrahim *et al*. (36), and Ghoneim *et al*. (37), Who reported that MDA levels were significantly elevated after tramadol exposure in some tissues. In the current research, intraperitoneal injection of tramadol (20 mg/kg) significantly decreased the intracellular GSH content in testicular tissue, compared with the control group. Same findings were recorded by Ghoneim *et al*. (37) and Sheweita *et al.* (38). The deficiency of the antioxidant system was evidenced by depletion of GSH levels in testicular tissue which in turn leads to a further increase in MDA levels. This condition can be followed by necrosis and apoptosis due to oxidation of the thiol group in the proteins of the mitochondrial membrane (39). Our results also revealed a significant decrease in SOD activity, which agreed with some previous studies (35, 36). The depletion of GSH and SOD indicates suppression of ROS scavenging capability. Our findings also showed NO levels in testicular tissue were increased, when compared with the control group. In parallel with our results, previous findings suggested that tramadol might induce testicular dysfunction due to the augmented production of NO and oxidative stress (40). Our results also revealed that intramuscular injection of boldenone (5 mg/kg) induced oxidative stress in testicular tissue which was indicated by a significant increase in MDA and NO and on the other hand a significant lowering of GSH and SOD in testicular tissue. In a previous study (41), the authors verified an elevation of MDA and No as well as a decrement of SOD and GSH in rabbits due to injection with boldenone; furthermore, a significant induction of lipid peroxidation, as well as complete inhibition of antioxidant capacity in testicular tissues in rats (42). 

Physiologically, testis undergoes programmed cell death (apoptosis) to get rid of damaged cells (43). On the other hand, it was reported that there is a significant relationship between apoptosis and male infertility (44). Two important molecules are implicated in cell death: Bax (proapoptotic) and Bcl2 (antiapoptotic) and their ratio decides if the cell will experience apoptosis and in addition, caspase3 represents the intrinsic pathway of apoptosis (45). Bcl2 can as well regulate the activity of caspase indirectly through heterodimerization of Bcl2 with Bax to prevent apoptosis (36), so cellular apoptosis is enhanced by dysregulation of Bcl2 or Bax (46). In the current study, our findings reported that injection of tramadol for 2 months resulted in a significant increase in Bax and a significant decrease in Bcl2 leading to an increase in Bax/Bcl2 ratio. Additionally, tramadol induced caspase3 activity. All these findings indicate the presence of cellular apoptosis in testicular cells. Our results were in agreement with (47) who recorded an up-regulation of Bax and caspase3 and a down-regulation of Bcl2. Similar findings were recorded in the rat cerebral cortex and lung tissues (48). Similarly, the intramuscular injection of boldenone for 2 months showed a significant dysregulation of apoptotic biomarkers; Bax, Bcl2, and caspase3 as recorded in tramadol injection, indicating the existence of apoptosis. In a previous study conducted on rabbits, results revealed that boldenone leads to activation of apoptosis in liver parenchyma and up-regulation of caspase activity (49). 

The reproductive efficiency of males and females is interrelated mainly to the hypothalamic-pituitary-gonadal axis so any interference in this axis might lead to reduced or complete infertility (50). Exposure of testes to heavy metals or any toxicants can cause damage to the mitochondria and down-regulate the expression of steroidogenic acute regulatory protein (StAR) and then inhibit the secretion of steroids. In the current study, injection of tramadol for 2 months had significantly reduced the serum levels of FSH, LH, and testosterone. A previous study conducted on tramadol showed similar results (51). These findings may be due to the interference of opioids with the normal release of GnRH at the level of the hypothalamus leading to the decrease of FSH, LH, and testosterone (52). Injection of boldenone, also for 2 months, revealed similar results as those obtained in groups injected with tramadol showing a significant reduction in FSH, LH, and testosterone levels in serum. These results are in agreement with those of Behairy *et al.* (2020) (41). We also reported a significant down-regulation of StaR and HSD17B3 expression after injection of both tramadol and boldenone. Reduction of steroid levels, as well as down-regulation of StaR and HSD17B3, may occur due to elevated levels of ROS and reduction of antioxidant capacity as SOD, glutathione peroxidase (53), and finally leading to down-regulation of StaR and HSD17B3 (45). The mixture of tramadol and boldenone were injected into rats for the first time to scout their additive effect because nowadays both drugs are being concurrently abused especially among youth. We found that the mixture showed significant detrimental effects in most of the parameters as compared with the groups administered the drugs alone.

**Table 1:** Gene-specific primer pairs for StAR, 17β HSD, and β actin

	** Primer sequence**
StAR	Forward primer :5’- CGAAACCCTAGTCAGGCACA -3 Reverse primer:5’- - TGTCTCACTGTGGTCCAAGC -3
17 B HSD	Forward primer: 5-TTCTGCAAGGCTTTACCAGG-3 Reverse primer: 5-ACAAACTCATCGGCGGTCTT-3
beta-actin	Forward primer 5′-TGTTTGAGACCTTCAACACC-3′ Reverse primer 5′-CGCTCATTGCCGATAGTGAT-3′

**Table 2:** Effect of IP injection of tramadol and IM injection of boldenone alone and in combination on oxidative stress biomarkers in testicular tissue

**Groups**	**MDA** (nmol** mg protein)**	**GSH** **(Mmol/mg protein)**	**SOD** ** (U / mg protein)**	**NO** **(nmol/mg protein)**
**Control **	41.33±7.6	±58.400.57	43.97±5.57	15.66±1.34
**TRAM 20 mg/kg**	107.6±6.65^a^	±29.43.73^a^	21.84±2.05^a^	42.52±1.89^a^
**BOLD 5 mg/kg**	105.68±7.74^a^	±30.042.26^a^	22.92±2.11^a^	42.7±4.63^a^
**TRAM 20 mg/kg + BOLD 5 mg/kg**	142.12±9.67^abc^	±19.622.15^abc^	14.28±2.5^abc^	69.6±5.47^abc^

Data are expressed as (mean ± SE). Statistics were done by one-way ANOVA and Tukey's test

TRAM: Tramadol, BOLD: Boldenone, MDA: Malondialdehyde, GSH: Reduced glutathione, SOD: Superoxide dismutase, NO: Nitric oxide, IP: Intraperitoneal, IM: Intramuscular

a *P*<0.05: Statistically significant from the control group

b *P*<0.05: Statistically significant from Tramadol

c *P*<0.05: Statistically significant from Boldenone

**Table 3:** Effect of IP injection of tramadol and IM injection of boldenone alone and in combination on apoptotic biomarkers in testicular tissue

**Groups**	**Bax** **(pg/mg protein)**	**Bcl2** **(pg/mg protein)**	**Bax/Bcl2 ratio**	** Casp -3** **(ng/mg protein)**
**Control **	115.62±1.85	±140.466.28	0.82	1.68±0.2
**TRAM 20 mg/kg**	258.88±13.88^a^	±62.987.71^a^	4.11(501.2%)	4.9±0.61^a^
**BOLD 5 mg/kg**	256.44±14.41^a^	±67.51.1^a^	3.9(475.6%)	5.12±0.42^a^
**TRAM 20 mg/kg + BOLD 5 mg/kg**	293.62±10.33^abc^	±47.462.15^abc^	6.19(754.8%)	6.76±0.26^abc^

Data are expressed as (mean ± SE). Statistics were done by one-way ANOVA and Tukey's test

TRAM: Tramadol, BOLD: Boldenone, Bax: Bcl2 Associated X, Apoptosis Regulator, Bcl2: B-cell lymphoma 2, IP: Intraperitoneal, IM: Intramuscular

a *P*<0.05: Statistically significant from the control group

b *P*<0.05: Statistically significant from Tramadol

c *P*<0.05: Statistically significant from Boldenone

**Table 4:** Effect of IP injection of tramadol and IM injection of boldenone alone and in combination on hormonal changes in serum

**Groups**	**LH** **(mIU/ml)**	**FSH** **(mIU/ml)**	**Free testosterone** **(pg/ml)**
**Control **	5.48±0.4	±5.260.2	69.5±0.79
**TRAM 20 mg/kg**	3.62±0.09^ac^	±3.220.11^ac^	31.2±0.2^ac^
**BOLD 5 mg/kg**	2.29±0.05^ab^	±2.560.09^ab^	22.9±0.36^ab^
**TRAM 20 mg/kg + BOLD 5 mg/kg**	1.8±0.06^abc^	±1.940.03^abc^	17.5±0.14^ab^

Data are expressed as (mean ± SE). Statistics were done by one-way ANOVA and Tukey's test

TRAM: Tramadol, BOLD: Boldenone, LH: Luteinizing hormone, FSH: Follicle-stimulating hormone, IP: Intraperitoneal, IM: Intramuscular

a *P*<0.05: Statistically significant from the control group

b *P*<0.05: Statistically significant from Tramadol

c *P*<0.05: Statistically significant from Boldenone

TRAM: Tramadol, BOLD: Boldenone, Comb: Combination, StAR: Steroidogenic acute regulatory gene, 17β HSD: 17β-hydroxysteroid dehydrogenase

## Conclusion

In summary, we have illuminated the possible underlying mechanisms beyond tramadol and boldenone-induced testicular dysfunction when used for 2 months which might be due to ROS release as well as inhibiting of antioxidant defense mechanisms. ROS released due to Tram and Bold injection not only affected the integrity of the testicular cells but also played a detrimental role in the down-regulation of StaR and HSD17β3 genes which interfered with the release of steroids at normal levels. We also concluded the implication of these drugs alone or in combination in inducing apoptosis in testicular tissue via elevation of Bax/Bcl2 ratio and caspase 3 levels. Unfavorable histological injury on the testicular tissue also has been verified. Finally, we concluded that the use of these drugs in combination has the worst effect.

## Authors’ Contributions

NAM and MEAE Conceived and designed the experiments. NAM, SK, GFA, and MEAE Contributed reagents/ materials/analysis tools. NAM, GFA, MEAE, and SK Performed the experiments, analyzed the data, prepared figures, and tables, and wrote and reviewed the manuscript. All authors read and approved the final version of the manuscript. 

## Ethical Approval

Experimental protocol followed the Ethics and Animal Care Committee of the National Research Centre and the recommendations of the National Institutes of Health Guide for Care and Use of Laboratory Animals (NIH Publications No. 8023, revised 1978). 

## Conflicts of Interest

The authors declare no conflicts of interest.
